# Factors associated with cholestasis in newborns with gastroschisis

**DOI:** 10.1590/1984-0462/2024/42/2022152

**Published:** 2023-07-10

**Authors:** Juliana Zoboli Del Bigio, Ana Cristina Aoun Tannuri, Mário Cícero Falcão, Felipe Yu Matsushita, Werther Brunow de Carvalho

**Affiliations:** a Universidade de São Paulo, São Paulo, SP, Brazil.

**Keywords:** Gastroschisis, Parenteral nutrition, Cholestasis, Newborn infant, Gastrosquise, Nutrição parenteral, Colestase, Recém-nascido

## Abstract

**Objective:**

To describe the incidence and to analyze risk factors associated with cholestasis in neonates with gastroschisis.

**Methods:**

This is a retrospective cohort study in a tertiary single center analyzing 181 newborns with gastroschisis between 2009 and 2020. The following risk factors associated with cholestasis were analyzed: gestational age, birth weight, type of gastroschisis, silo closure or immediate closure, days of parenteral nutrition, type of lipid emulsion, days of fasting, days to reach a full diet, days with central venous catheter, presence of infections, and outcomes.

**Results:**

Among the 176 patients evaluated, 41 (23.3%) evolved with cholestasis. In the univariate analysis, low birth weight (p=0.023), prematurity (p<0.001), lipid emulsion with medium-chain triglycerides and long-chain triglycerides (p=0.001) and death (p<0.001) were associated with cholestasis. In the multivariate analysis, patients who received lipid emulsion with fish oil instead of medium chain triglycerides/long chain triglycerides (MCT/LCT) emulsion had a lower risk of cholestasis.

**Conclusions:**

Our study shows that lipid emulsion with fish oil is associated with a lower risk of cholestasis in neonates with gastroschisis. However, this is a retrospective study and a prospective study should be performed to confirm the results.

## RESUMO

**Objetivo:**

Analisar a incidência e os fatores de risco associados à colestase em recém-nascidos com gastrosquise.

**Métodos:**

Estudo de coorte retrospectivo em um único centro terciário, que analisou 181 recém-nascidos com gastrosquise entre 2009 e 2020. Foram examinados os seguintes fatores de risco associados à colestase: idade gestacional, peso ao nascer, tipo de gastrosquise, fechamento com silo ou fechamento imediato, dias de uso nutrição parenteral, tipo de emulsão lipídica, dias de jejum, dias para atingir a dieta completa, dias com cateter venoso central, presença de infecções e desfechos.

**Resultados:**

Dos 176 pacientes avaliados, 41 (23,3%) evoluíram com colestase. Baixo peso ao nascer (p=0,023), prematuridade (p<0,001), emulsão lipídica com triglicerídeos de cadeia média e triglicerídeos de cadeia longa (p=0,001) e óbito (p<0,001) foram associados à colestase. Na análise multivariada, os pacientes que receberam emulsão lipídica com óleo de peixe em vez da emulsão diária de triglicérides de cadeia média/triglicérides de cadeia longa (MCT/LCT) apresentaram menor risco de colestase.

**Conclusões:**

Nosso estudo mostra que a emulsão lipídica com óleo de peixe está associada a menor risco de colestase em neonatos com gastrosquise, porém este é um estudo retrospectivo, e um estudo prospectivo deve ser realizado para confirmar os resultados.

## INTRODUCTION

Gastroschisis is a birth defect of the abdominal wall. In most cases the defect is on the right side of the umbilical cord insertion, being rarely on the left.^1^ The incidence of gastroschisis has been increasing worldwide since 1995 and it is about 4.42 cases for every 10.000 live births).^2–4^

Gastroschisis can be classified as simple, when the defect is isolated, and complex, when the defect is associated with other intestinal anomalies such as intestinal atresia, intestinal perforation, necrotic segments or volvulus.^3–5^ Complex gastroschisis, prematurity, and low birth weight are associated with worse clinical outcomes, with higher mortality (<5% in large centers), a higher incidence of sepsis and catheter-related infections, cholestasis, short bowel syndrome, longer time to achieve full enteral nutrition, longer parenteral nutrition (PN) time and longer hospital stays.^6^

After surgical correction of gastroschisis, a period of intestinal hypomotility usually occurs and the etiology of the intestinal dysfunction has yet to be clarified.^7,8,9,10^ Prolonged use of PN is associated with significant complications, high morbidity, and potential mortality. Intestinal failure-associated liver disease (IFALD) is the most prevalent complication affecting children who receive long-term PN.^11,12^ Since it is multifactorial, there are several proposals to explain IFALD: prematurity, time to reach full enteral nutrition, type of lipid emulsion, the presence of multiple infections, and intestinal dysbiosis.^13^

The lipid emulsions medium-chain triglycerides/long-chain triglycerides (MCT/LCT) contain high concentrations of ω-6 polyunsaturated fatty acids (PUFA) such as linoleic acid, and relatively low concentrations of α-tocopherol that it exhibits strong antioxidant effects compared with the lipid emulsions containing fish oil.^11^ Lipid emulsions based purely on vegetable oils also contain higher concentrations of phytosterols compared with other lipid sources (e.g., those containing fish oil).^14^ There is evidence that phytosterols may contribute to the development of IFALD, though the role of phytosterols in the development of IFALD remains controversial.^15^ Lipid emulsions containing fish oil offer several advantages compared with those containing only soybean oil, including high concentrations of the ω-3 PUFA (docosahexaenoic acid and eicosapentaenoic acid), and the antioxidant α-tocopherol, reduced ω-6 PUFA content, and a reduced phytosterol load.^15^

Knowing that gastroschisis is a congenital malformation that can occur with prolonged fasting, use of PN and infection, it becomes important to assess the incidence of cholestasis and the factors that may be related.

The objectives of the study included reporting the overall incidence of cholestasis in this specific population as well as the risk factors for it. Among these factors, we relate some inherent to gastroschisis such as the use of a silo, the need for reoperation and others common to cholestasis such as fasting and use of PN. We also emphasize the concern to report the role of lipid alone in the incidence. A multivariate analysis was performed evaluating the role of lipids in PN in the incidence of IFALD.

## METHOD

This is an observational, retrospective, cohort study conducted in newborns with gastroschisis treated at the Neonatal Intensive Care Center of the Child and Adolescent Institute, Hospital das Clínicas, Faculdade de Medicina, Universidade de São Paulo, Brazil, between January 2009 and December 2020. The study was approved by the Ethics Committee of the Department of Pediatrics and the Ethics Committee for Analysis of Research Projects of the institution, protocol no. 2,476,188.

The inclusion criteria was the diagnosis of gastroschisis, and the exclusion criteria was the development of short bowel syndrome. In order to carry out the Cox model, newborns who received the lipid emulsion twice a week were excluded.

Regarding nutrition, in our unit, PN is started on the first day of life for hemodynamically stable newborns in infants with gastroschisis. Since May 2016, the lipid emulsion of choice has been a mixture with fish oil. Before 2016, lipid emulsion with MCT/LCT was used twice a week (from January 2009 to March 2012) and then daily (from April 2012 to April 2016). The lipid emulsion SMOF 20% has the following components and proportions: soybean oil (6%), MCT (6%), olive oil (5%), fish oil (3%) and soy-based emulsion containing 50/50% MCT/LCT. In PN, we usually use a dose of 3 g/kg/day of lipid as well as amino acids and glucose infusion rate according to blood glucose. Standardized in PN, we use trace elements, selenium and multivitamins.

All data was collected from medical records. The following epidemiological and clinical variables were analyzed by dividing groups according to the presence of cholestasis: gestational age (weeks); birth weight (grams); type of gastroschisis (simple or complex); silo closure or immediate closure; reoperation, fasting time (days); type of lipid emulsion (MCT/LCT 20% or mixed lipid based emulsion with fish oil); total time of PN (days); time to achieve full enteral nutrition (days) (considering the value of 120 mL/kg/day); length of hospitalization (days); infection and outcomes (discharge or death).

The laboratory variables analyzed were central and peripheral blood culture, catheter culture, urine culture and direct bilirubin (DB) in (mg/dL). Cholestasis was defined as presence of direct bilirubin (DB) greater than 2 mg/dL^12,13^ at any time of hospitalization.

Results are presented in proportions, means with standard deviation or medians with interquartile range. Continuous variables were tested for normality using Kolmogorov-Smirnov test. Comparisons between groups were performed using Mann-Whitney U test for continuous variables and chi-square for categorical variables.

Cholestasis was analyzed using Cox’s proportional regression. The results are expressed in terms of hazard ratio (95% confidence interval) and adjusted for potential confounders for cholestasis (reoperation, fasting duration, sepsis, central venous access duration, time to achieve full enteral nutrition, and total PN duration). Potential confounders and models were identified using a theoretical framework through direct acyclic graphs (DAG).

All analyses were performed using IBM Statistical Package for the Social Sciences (SPSS) Statistics for Macintosh, version 25.0. A p-value lower than 0.05 was considered significant.

## RESULTS

A total of 181 patients with gastroschisis between 2009 and 2020 were enrolled. Among these, five evolved with short bowel syndrome and were excluded from the study, and 49 received the lipid emulsion twice a week.

Of the 176 newborns, 53.4% were male and there was a predominance of preterm infants (56.8%). Complex gastroschisis was present in 15.3% of patients (27 cases), with a reoperation rate of 25.6%. During the first surgical approach it was necessary to use the silo in 15.9% of the cases, most of them being reoperated within seven days. Bloodstream infections occurred in 36.9% of newborns and the survival rate was greater than 90% (Table 1).

**Table 1.** Demographic data of the 176 newborn infants with gastroschisis.

	n=176 (%)
Sex	
Male	94 (53.4)
Preterm (<37 weeks)	
Yes	100 (56.8)
Type of gastroschisis	
Simple	149 (84.7)
Reoperation	
Yes	45 (25.6)
Silo closure	
Yes	28 (15.9)
Blood culture positive	
Yes	65 (36.9)
Outcome	
Discharged	159 (90.3)
Death	17 (9.7)

Median birth weight was 2320 grams, median fasting was 22 days and median time of PN was 27 days. The median time to reach a full enteral diet was 27 days, ranging from 21 to 40. Regarding the length of hospital stay, the median was 35 days.

Table 2 shows, through univariate analysis, the clinical characteristics of these newborns with gastroschisis in the groups with and without cholestasis. All analyzed variables are associated with cholestasis, except for the use of the silo. Among the 176 patients evaluated, 41 evolved with IFALD (23.3%). Newborns with cholestasis had a lower birth weight (p=0.023) and gestational age (p<0.001). Cholestasis was more frequent in those who had complex gastroschisis (p=0.005) and who were reoperated (p=0.002). Regarding nutritional factors, the median time of fasting (p <0.001), PN (p<0.001), and to reach the full diet (p<0.001) were higher in the group with cholestasis. In addition, the group with daily lipids also had more cholestasis (p<0.001). Cholestasis was also associated with infection (p<0.001) and length of stay in the intensive care unit (p<0.001).

**Table 2.** Comparison between clinical characteristics and cholestasis in neonates with gastroschisis (univariate analysis).

	Cholestasis (n=41)	No cholestasis (n=135)	p-value
Birth weight (grams)*	2,075 (1,775–2,510)	2,370 (1,990–2,610)	0.023^‡^
Gestational age (weeks)*	34.8 (34–36.3)	36.8 (35.9–37.2)	<0.001^‡^
Complex gastroschisis^†^	12 (29.2%)	15 (11.1%)	0.005^§^
Reoperation^†^	18 (43.9%)	27 (20.0%)	0.002^§^
Silo closure^†^	8 (19.5%)	20 (14.8%)	0.471^§^
Days of fasting*	33 (22–59)	20 (15–29)	<0.001^‡^
Days of total parenteral nutrition*	35 (26–68)	25 (20–36)	<0.001^‡^
Days to achieve full enteral nutrition *	43 (28–93)	27 (22–35)	<0.001^‡^
Days of central venous access*	42 (30–80)	29 (24–41)	<0.001^‡^
Positive blood culture^†^	13 (31.7%)	37 (27.4%)	<0.001^§^
Days in the NICU duration, median (IQR)	45 (30–84)	32 (26–42)	<0.001^‡^
Death	12 (29.2%)	5 (3.7%)	<0.001^§^
Soy-based emulsion infusion daily	19 (46.3%)	28 (20.7%)	0.001^§^

Data expressed in *median (interquartile range — IQR) or in ^†^N (%); ^‡^Mann-Whitney test; ^§^Chi-square; NICU: Neonatal Intensive Care Unit; IQR: interquartile range.

Table 3 shows, through Cox’s proportional regression in terms of hazard ratio (95% CI), the association between daily lipid infusion and cholestasis in neonates with gastroschisis, excluding newborns who received lipids only twice a week. Daily lipid emulsion MCT/LCT 20% was associated with higher cholestasis risk compared to lipid emulsions containing fish oil even after adjusting for potential confounders (reoperation, fasting duration, sepsis, central venous access duration, time to achieve full enteral diet and total PN duration).

**Table 3.** Association between soy-based lipid infusion and cholestasis in neonates with gastroschisis using Cox’s proportional regression in terms of hazard ratio (95% confidence interval).

Association analysis	Hazard ratio	p-value	95% CI
Model 1	2.73	0.006	1.34–5.57
Model 2	2.32	0.023	1.12–4.8
Model 3	2.55	0.017	1.18–5.49
Model 4	2.35	0.023	1.12–4.89
Model 5	4.18	0.002	1.70–10.24
Model 6	2.45	0.016	1.18–5.08

Model 1: univariate analysis (soy-based emulsion *vs*. mixed lipid-based emulsion with fish oil); Model 2: adjusted for reoperation and fasting duration; Model 3: adjusted for sepsis and fasting duration; Model 4: adjusted for central venous access duration and fasting duration; Model 5: adjusted for time to achieve full enteral diet; Model 6: adjusted for total parenteral nutrition duration. CI: confidence interval.

## DISCUSSION

The incidence of cholestasis was 23.3%, considering direct bilirubin (DB) >2 mg/dL in any measure during hospitalization; similar to that found by Fallon et al.: 27.6%.^16^

It is known that neonatal cholestasis can be multifactorial, as in the case of newborns with gastroschisis. The need for the time of delivery with a gestational age closer to 37 weeks, low birth weight, the need for prolonged fasting and PN due to the dysmotility of the pathology, the use of a central venous catheter, the possibility of surgical re-approach and the presence of complex gastroschisis make this condition an interesting model to analyze cholestasis. Our model suggests that soy-based intralipids are associated with higher cholestasis risk compared to lipid emulsions containing fish oil, even after adjusting for potential confounders (reoperation, fasting duration, sepsis, central venous access duration, time to achieve full enteral diet and total PN duration). Of the 181 newborns studied, five evolved to short bowel and were excluded from the analysis. Ninety-four newborns were male, 56.8% were premature and 25.6% required surgical re-approach. Compared to Raymond and other studies, prematurity had a similar rate (56.3%). Mortality was 9.7%, a little higher than that found by Raymond et al.,^6^ but lower than those found in other Brazilian regions. A study conducted in Belo Horizonte (MG) showed a mortality rate of 14.9%.^17^

Low birth weight (p<0.023) and prematurity (p<0.001) were associated with cholestasis. The increased incidence of cholestasis in the most premature infants is likely to owe in part to the functional immaturity of the hepatobiliary system. Compared with full-term infants, preterm neonates are inefficient in processing bile acids.^18^

Cairo et al. found an incidence of cholestasis of almost 80% in newborns with gastroschisis, but using direct bilirubin greater than 1 mg/dL and also showed that all patients improved cholestasis with initiation of enteral feeds.^19^

The presence of prolonged fasting, PN, the lipid emulsion used and infections, as well as other factors, play a role in the etiology of IFALD. PN in the setting of intestinal dysmotility and metabolic and infectious complications remains a major cause of morbidity and mortality in the gastroschisis population.^16^ In this study, we demonstrated that cholestasis was associated with almost all studied parameters: birth weight, gestational age, complex gastroschisis, reoperation, fasting duration, total PN duration, time to achieve full enteral nutrition, central venous access duration, positive blood culture, Neonatal Intensive Care Unit duration, death, and daily intralipid. While Fallon et al.^16^ found increased duration of PN and increased incidence of cholestasis with silo placement compared to the primary closure of the abdominal wall defect, we did not observe this in our patients.

Lipid emulsions have evolved over the decades with the aim of reducing the risk and/or evolution of IFALD. IFALD was the primary cause of death of children with intestinal failure. While the pathophysiology of IFALD is multifactorial, much attention has been devoted recently to the critical role that ω-6 PUFA containing intravenous lipid emulsions (ILEs) play in the development of IFALD.^15^

The most current recommendation of the European Society for Paediatric Gastroenterology Hepatology and Nutrition — ESPGHAN 2018^11^ regarding lipid emulsion in PN for neonatal and pediatric use is to avoid the use of pure soy due to the high level of phytosterols and the use of a mixed emulsion with or without fish oil is recommended. In this study, we tried to find the risk factors for cholestasis and the role of the lipid emulsion as a unique factor. In the Cox regression, the use of lipid emulsion without fish was involved in the incidence of cholestasis.

A recent randomized, controlled pilot study showed that the use of lipid emulsion with fish oil reduces the risk of progressive IFALD in children with intestinal failure. Despite the small number of participants, the study is relevant for being a pilot in children. It is not possible to be sure that the benefit of lipid emulsion with fish oil is due to the presence of ω-3 or a lesser amount of ω-6.^15^

In this study, it was possible to state that the lipid emulsion with fish oil, when compared to the emulsion without ω-3, is associated with a lower IFALD (p=0.06). Despite being a retrospective study, the sample chosen is of a single, rare pathology and carried out in a single center, ensuring greater uniformity. Our study shows that lipid emulsion with fish oil is associated with lower risk of cholestasis in neonates with gastroschisis, compared to soy-based lipid emulsion.

Several important questions remain to be answered about the use of lipid in children with PN-dependent intestinal failure.^15,20,21^ In gastroschisis, despite the presence of confounding factors for IFALD such as low birth weight, gestational age, the presence of complex gastroschisis, among others, the lipid in NP plays an important role in its pathogenesis with a higher weight for emulsion without ω-3.

We know that the type of lipid emulsion was not randomized, and there is a limitation in that the neonates receiving MCT/LCT 20% are a historic group, thus the comparison is between a group treated before April 2016 with the group treated afterwards. Another limitation regards the fact that this is a unicentric study, but with a large number of subjects.

Studies in infants and children receiving long-term PN have shown that multicomponent lipid emulsions containing fish oil reduce the risk of cholestasis and improve biochemical measures of liver function.^15^

Future randomized controlled trials with a long-term follow-up are necessary to explore the strength of the evidence, especially in the neonatal period, where other factors also appear as confounders — such as low birth weight and prematurity.

## Data Availability

The database was made available in the Sigma system only for the main author (Juliana Zoboli Del Bigio), with the authorization of the responsible person.

## References

[1] Suver D, Lee SL, Shekherdimian S, Kim SS (2008). Left-sided gastroschisis: higher incidence of extraintestinal congenital anomalies. Am J Surg..

[2] Kirby RS, Marshall J, Tanner JP, Salemi JL, Feldkamp ML, Marengo L (2013). Prevalence and correlates of gastroschisis in 15 states, 1995 to 2005. Obstet Gynecol..

[3] Lakshminarayanan B, Lakhoo K (2014). Abdominal wall defects. Early Hum Dev..

[4] Vu LT, Nobuhara KK, Laurent C, Shaw GM (2008). Increasing prevalence of gastroschisis: population-based study in California. J Pediatr..

[5] Bergholz R, Boettcher M, Reinshagen K, Wenke K (2014). Complex gastroschisis is a different entity to simple gastroschisis affecting morbidity and mortality-a systematic review and meta-analysis. J Pediatr Surg..

[6] Raymond SL, Hawkins RB, Peter SD, Downard CD, Qureshi FG, Renaud E (2020). Predicting morbidity and mortality in neonates born with gastroschisis. J Surg Res..

[7] Arnold MA, Chang DC, Nabaweesi R, Colombani PM, Fischer AC, Lau HT (2007). Development and validation of a risk stratification index to predict death in gastroschisis. J Pediatr Surg..

[8] Lao OB, Larison C, Garrison MM, Waldhausen JH, Goldin AB (2010). Outcomes in neonates with gastroschisis in U.S. children’s hospitals. Am J Perinatol..

[9] Régis AC, Rojas-Moscoso JA, Gonçalves FL, Schmidt AF, Mónica FZ, Antunes E (2011). The cholinergic response is increased in isolated ileum from gastroschisis rat model. Pediatr Surg Int..

[10] Santos MM, Tannuri U, Maksoud JG (2003). Alterations of enteric nerve plexus in experimental gastroschisis: is there a delay in the maturation?. J Pediatr Surg..

[11] Lapillonne A, Mis NF, Goulet O, van den Akker CH, Wu J, Koletzko B (2018). ESPGHAN/ESPEN/ESPR/CSPEN guidelines on pediatric parenteral nutrition : lipids. Clin Nutr..

[12] Goulet OJ, Cai W, Seo JM (2020). Lipid emulsion use in pediatric patients requiring long-term parenteral nutrition. JPEN J Parenter Enteral Nutr..

[13] Lauriti G, Zani A, Aufieri R, Cananzi M, Chiesa PL, Eaton S (2014). Incidence, prevention, and treatment of parenteral nutrition-associated cholestasis and intestinal failure-associated liver disease in infants and children: a systematic review. JPEN J Parenter Enteral Nutr..

[14] Forchielli ML, Bersani G, Tala S, Grossi G, Puggioli C, Masi M (2010). The spectrum of plant and animal sterols in different oil-derived intravenous emulsions. Lipids..

[15] Diamond IR, Grant RC, Pencharz PB, Silva N, Feldman BM, Fitzgerald P (2017). Preventing the progression of intestinal failure-associated liver disease in infants using a composite lipid emulsion: a pilot randomized controlled trial of SMOFlipid. JPEN J Parenter Enteral Nutr..

[16] Fallon EM, Mitchell PD, Potemkin AK, Nehra D, Arsenault DA, Robinson EM (2012). Cholestasis and growth in neonates with gastroschisis. J Pediatr Surg..

[17] Alves FM, Miranda ME, Aguiar MJ, Viana MC (2016). Nutritional management and postoperative prognosis of newborns submitted to primary surgical repair of gastroschisis. J Pediatr (Rio J)..

[18] Satrom K, Gourley G (2016). Cholestasis in preterm infants. Clin Perinatol..

[19] Cairo SB, Osak AH, Berkelhamer SK, McLaughlin C, Rothstein DH (2019). Direct hyperbilirubinemia in newborns with gastroschisis. Pediatr Surg Int..

[20] Seida JC, Mager DR, Hartling L, Vandermeer B, Turner JM (2013). Parenteral ω-3 fatty acid lipid emulsions for children with intestinal failure and other conditions: a systematic review. JPEN J Parenter Enteral Nutr..

[21] Wales PW, Allen N, Worthington P, George D, Compher C, American Society for Parenteral and Enteral Nutrition (2014). A.S.P.E.N. clinical guidelines: support of pediatric patients with intestinal failure at risk of parenteral nutrition-associated liver disease. JPEN J Parenter Enteral Nutr..

